# Final height and body mass index in adult survivors of childhood acute lymphoblastic leukemia treated without cranial radiotherapy: a retrospective longitudinal multicenter Italian study

**DOI:** 10.1186/1471-2431-14-236

**Published:** 2014-09-22

**Authors:** Patrizia Bruzzi, Barbara Predieri, Andrea Corrias, Alberto Marsciani, Maria Elisabeth Street, Aurora Rossidivita, Paolo Paolucci, Lorenzo Iughetti

**Affiliations:** Pediatric Unit, Department of Medical and Surgical Sciences for Mothers, Children and Adults, University of Modena & Reggio Emilia, Via del Pozzo, 71, 41124 Modena, Italy; Regina Margherita Children’s Hospital, Department of Pediatric Endocrinology and Diabetology, University of Torino, Piazza Polonia, 94, 10126 Torino, Italy; Pediatric Department, Infermi Hospital, viale Luigi Settembrini, 2, 47900 Rimini, Italy; Department of Pediatrics, University Hospital of Parma, via Gramsci, 14, 43126 Parma, Italy; Pediatric Department, Cattolica University, Largo Agostino Gemelli, 8, Roma, Italy

## Abstract

**Background:**

Young adult survivors of childhood acute lymphoblastic leukemia (ALL) treated with protocols including cranial radiotherapy demonstrate a persistent weight gain and reduced final height. Published reports on the effects on growth of different oncologic therapies are conflicting and difficult to interpret because they combined children treated with both cranial irradiation and multi-agent chemotherapy. Our study investigated the effect of chemotherapy alone on body mass index (BMI) and on growth at the achievement of final height in a homogeneous cohort of Italian childhood ALL survivors.

**Methods:**

We retrospectively studied 162 Caucasian patients treated on the Italian Association of Pediatric Hematology and Oncology protocols without radiotherapy between 1989 and 2000 at five Italian centers with 107 inclusions (58 males). Height- and BMI-standard deviation score (SDS) were collected at diagnosis of ALL, at the end of treatment and at the achievement of final height. Changes in height SDS and BMI SDS with time were analyzed using dependent sample Student's t-test.

**Results:**

A significant reduction of height-SDS was documented during treatment in both genders. This reduction of height-SDS was not followed by an appropriate catch-up growth, despite the achievement of a mean final height within the normal range. At diagnosis females showed a lower mean BMI-SDS than males. During treatment, in the whole population, BMI-SDS increased significantly. After it, while males lost BMI-SDS, females showed its persistent increase.

**Conclusions:**

Survivors of childhood ALL generally seemed to achieve a normal final height with a BMI within the normal range. These parameters appeared to be only minimally affected by chemotherapy. Nevertheless, height catch-up growth was not completed after chemotherapy in both genders and all patients experienced an increase of BMI-SDS during chemotherapy that only females seemed to conserve until the achievement of final height.

## Background

Acute lymphoblastic leukemia (ALL) is the most common malignancy in children and represents 80% of all leukemia cases [1]. Recent progress in risk-adapted treatment for childhood ALL has secured eight-year event-free survival rates of approximately 90% [2–4]. Consequently, the late side-effects of the cancer treatment have obtained an increased attention and the long-term monitoring of survivors has become an important part of their overall health care [5]. Treatment of ALL during childhood is associated with final height deficit. Young age at diagnosis and radiotherapy are considered the major risk factors [6]. Moreover, adult survivors of childhood ALL have a four-fold excess risk of mortality from cardiovascular disease. Obesity seems highly prevalent among female and survivors treated with radiotherapy [7].

Nowadays, ALL survivors form the largest group of long-term survivors from childhood cancer, even if they are a heterogeneous group considering therapeutic protocols. In last decades, in most of them, cranial radiotherapy has been replaced by intrathecal chemotherapy, as standard central nervous system prophylaxis and treatment, and a reduced percentage of children have received cranial radiation [8] with a consequent reduction of sequelae related to this treatment modality. However, it has been suggested that also chemotherapy can negatively affect growth and endocrine functions [9, 10]. Published reports on the effects on growth of these therapies are conflicting and difficult to interpret because most studies have analyzed growth in children treated with both cranial irradiation and combination chemotherapy and disentangling the adverse contribution of these two major therapeutic modalities has proved tricky [11–13].

To evaluate the impact on growth, we performed a retrospective multicenter study in a large pediatric ALL population treated without radiotherapy, followed until the achievement of final height.

## Methods

### Design and setting

This study was a longitudinal, retrospective and multicenter study: we reviewed the clinical notes of patients treated for ALL at five Italian pediatric oncologic centers (Modena, Turin, Rome, Rimini and Parma) on Italian Association of Pediatric Hematology and Oncology (AIEOP) protocols, without radiotherapy, from 1989 to 2000, and followed until the achievement of adult height. Inclusion criteria comprised the successful completion of the treatment (standard risk protocols) and a continued first remission. Exclusion criteria included: relapse, exposure to radiotherapy and/or bone marrow transplant, the presence of other diseases potentially influencing growth (i.e. Down’s syndrome, neurofibromatosis type 1) and of chronic treatment with any other medication apart from the AIEOP protocols which might affect growth (i.e. growth hormone therapy, sex steroid) and lack of complete auxological data. Details concerning ALL treatment protocols were published elsewhere [14] and summarized in Table 1.

**Table 1:** **Characteristics of treatment protocols**

Protocols	87	8801	8802	8805	9101	9102	9501 – 9502 armA	9502 armB	2000
**PDN**	2500	1830	1830	150	2030	1970	1970	1620	1910
**VCR**	20.5	9	12	9	12	12	12	15	18
**DNM**	180	160	160	-	120	120	-	120	120
**MTX IT**	٧	٧	٧	٧	٧	٧	٧	٧	٧
**L-ASP**	5400	80000	80000	-	80000	80000	120000	120000	80000
**6-MP**	6000	29920	25900	-	5100	28920	27350	6710	6830
**MTX IV**	180	1540	1500	30	1488	1500	1488	1500	1500
**CPM**	-	2000	3000	1600	1000	3000	1000	3000	2500
**DESA**	-	189	249	300	249	249	261	297	409
**6-TG**	-	900	900	-	840	840	780	780	780
**ARA-C**	-	600	600	900	600	1800	600	1600	600-1800
**IFO**	-	-	-	12000	-	-	-	-	-
**VM-26**	-	-	-	600	-	-	-	-	-
**ADM**	-	60	120	150	120	120	120	120	60-120
**Num. patients exposed**	2	1	2	4	5	28	2	57	6

Legend: Drugs are expressed as cumulative dose (mg/mq). PDN, prednisolone; VCR, vincristine; DNM, daunorubicin; MTX, metotrexate; IT, intrathecal; ٧ included; L-ASP; L-asparaginase (UI/mq); 6-MP, 6-mercatopurine; IV, intravenously; CPM, cyclophosphamide; DESA, dexamethasone; 6-TG, 6-thioguanine; ARA-C, cytosine arabinoside.; IFO, ifosfamide; VM-26,teniposide; ADM, adriamycin.

Provincial Ethical Committee approved the protocol study (practice 185/11).

### Data collection

Age, height and weight at diagnosis of ALL (sT), at the end of treatment (EoT), and at final height (FH) were extracted from the record of each eligible patient. Uniformly, in every center, height was measured to the nearest 0.1-cm with a wall-mounted stadiometer (Harpenden, Crymych; UK); body weight was measured to the nearest 0.1-kg and body mass index (BMI) was obtained from the weight in kg/height in meters squared and expressed as standard deviation-score (SD-S) with respect to chronological age. The auxological instruments were routinely checked and calibrated. Height- and BMI- SDS were calculated for each value using age- and sex- specific World Health Organization (WHO) growth chart 2007 [15]. Parental height was also collected to estimate target height (TH), calculated according to the formula: [(mother’s height +13) + father’s height]/2 in males and [(mother’s height - 13) + father’s height]/2 in females [16]. Final height (FH) was defined as the standing height achieved when the linear growth velocity during the preceding year was less than 1 cm/year.

For each participant, demographic and therapeutic information was obtained, including gender, ethnicity, diagnostic white cell count (WBC) at sT and treatment modalities.

### Data analysis

All results, apart from ages expressed by median, were reported as the mean ± SD. Parametric statistical analysis (STATISTICA™ software, StatSoft Inc., Tulsa, OK, USA) was performed using dependent sample Student’s t-test to detect mean changes in height SDS and BMI SDS from diagnosis of ALL to final height.

Data were also analyzed according to gender and to groups of age (younger or older than four years at diagnosis). Independent t-test was performed to detect differences between groups. Gender, WBC, age at diagnosis, height at diagnosis and BMI at diagnosis, genetic target height were analyzed in a univariate regression model with final height SDS as dependent variable through Pearson’s correlation. Potential predictors of final height SDS and BMI SDS at FH were analyzed by multivariate regression models.

A P value below 0.05 was considered statistically significant.

## Results

Of 162 Caucasian patients treated on AIEOP ALL87, 88, 91, 95 and 2000 protocols without radiotherapy from 1989 to 2000, 107 met inclusion criteria. Causes of exclusion enclosed: relapse (7.4% of initial population), exposure to radiotherapy (6.7%) and lack of data (19.7%). None was excluded because of the presence of other diseases or of other chronic treatment potentially influencing growth. Only auxological parameters at sT were available in the 32 non-participants patients excluded because of lacking data. No statistical differences were detected between included and excluded patients among available variables (Table 2).

**Table 2:** **Meadian age (years; range); Height-SDS (media ± SDS); Height-SDS adjusted according to target height (media ± SDS) and BMI-SDS (media ± SDS) in study population and non-participants and according to gender from sT to FH**

	Total participant population	Participants males (58 pts)	Participant females (49 pts)	Non-participans males (17 pts)	Non-participans females (15 pts)
**Meadian age**					
**sT**	5.57 (1.20 – 13.73)	5.93 (1.31 – 12.85)	5.15 (1.20 – 13.73)	5.84 (1.15 – 12.74)	5.12 (1.14 – 13.45)
**EoT**	7.60 (3.0 – 15.98)	7.97 (3.23 – 14.97)	7.16 (3.0 – 15.98)	/	/
**FH**	17.52 (14.05 – 23.98)	17.68 (14.05 – 23.98)	17 · 24 (14.05- 23.7)	/	/
**Height-SDS**					
**sT**	0.61 ± 1.04	0.62 ± 1.05	0.60 ± 1.05	0.60 ± 1.02	0.62 ± 1.09
**EoT**	0.25 ± 1.08 (0.000)	0.30 ± 1.09 (0.003)	0.19 ± 1.09 (0.000)	/	/
**FH**	0.18 ± 1.13 (0.000)	0.27 ± 1.03 (0.013)	0.07 ± 1.25 (0.000)	/	/
**Height-SDS adjusted TH**					
**sT**	0.45 ± 1.22	0.51 ± 1.38	0.38 ± 1.22	/	/
**EoT**	0.04 ± 1.2 (0.000)	0.18 ± 1.36 (0.043)	-0.12 ± 1.11 (0.000)	/	/
**FH**	0.15 ± 1.20 (0.000)	0.37 ± 1.23	-0.13 ± 1.11 (0.022)	/	/
**BMI-SDS**					
**sT**	0.15 ± 1.32	0.39 ± 1.30	-0.12 ± 1.31†	0.35 ± 1.24	-0.09 ± 1.20
**EoT**	0.58 ± 1.34 (0.000)	0.82 ± 1.29 (0.010)	0.29 ± 1.36 (0.015) †	/	/
**FH**	0.51 ± 1.03 (0.010)	0.49 ± 1.09	0.54 ± 0.96 (0.001)	/	/

Only significant p-values versus sT are expressed into brackets. † Significant p-values Males versus Females (BMI-SDS sT: males vs females: p 0.0427; EoT: males vs females: p 0.0431).

Demographic data of participants and non-participants are shown in Table 2.

Among participants, there was no significant difference between male and female groups in age. The median time from diagnosis to assessment of FH was 11.90 ± 3.26 years (range 4.23 -18.46).

Table 2 lists anthropometric data over time.

**Figure 1:**
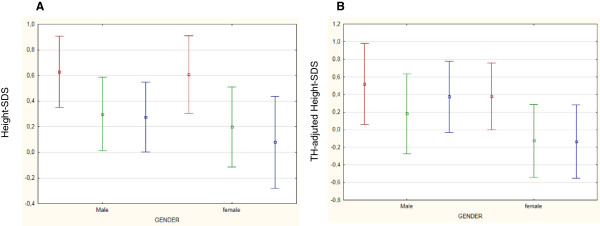
**Mean Height-SDS (A) and mean height-SDS adjusted according to TH (B) changes with time in both gender.** Legend: Data at diagnosis of ALL (sT) are colored in red. Data at the end of treatment (EoT) are colored in green. Data at final height (FH) are colored in blue.

**Figure 2:**
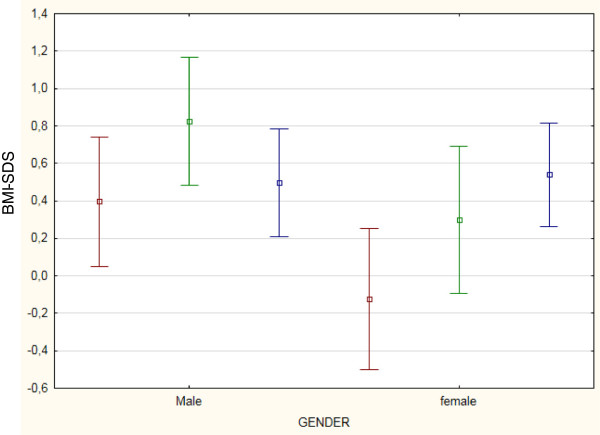
**Mean BMI-SDS changes with time in both gender.** Legend: Data at diagnosis of ALL (sT) are colored in red. Data at the end of treatment (EoT) are colored in green. Data at final height (FH) are colored in blue.

At every step-time, both males and females had a mean height- and BMI-SDS within the normal range (Figures 1 and 2). At sT, children appeared generally well-nourished, showing a mean SDS of + 0.61 for height and +0.15 for BMI. Only one girl presented a height-SDS less than -2 SD, but appropriate for her TH SDS (corresponding to -2.70 SD). Analyzing BMI SDS distribution at sT, 7 children were thin (BMI SDS less than -2 SD) and the same number were obese (BMI SDS more than +2 SD).

### Changes with time in height-SDS

In the whole population, a significant reduction of height-SDS was documented during treatment (from sT to EoT) in both genders (Table 2). This reduction of height-SDS was not followed by an appropriate catch-up growth (Figure 1). In fact, after the end of chemotherapy, height-SDS further decreased both in females and in males.

TH-SDS was 0.02 ± 1.10 SDS in the whole group, -0.08 ± 0.79 SDS in males and 0.16 ± 1.40 in females. At sT, height-SDS adjusted according to TH was higher than at EoT and at FH (Table 2). The discrepancy in height-SDS from TH-SDS reduced over time (TH SDS – sT SDS *vs* TH SDS – FH SDS: -0.59 *vs.* -0.16; -0.70 *vs.* -0.35; -0.44 *vs.* -0.09 in the whole population, males and females; respectively). Only females achieved a FH SDS significantly reduced than data at sT (Table 2).

**Figure 3:**
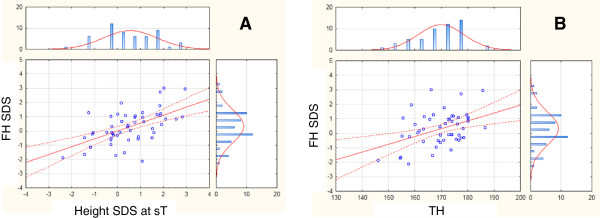
**Significant correlations between FH and height SDS at diagnosis (A) (r = 0.571, p = 0.018) and between FH and TH (B) (r = 0.410, p = 0.000) in the whole population.**

In the whole population FH-SDS correlated with height-SDS at sT (Figure 3A) and TH-SDS (Figure 3B).

### Changes with time in BMI-SDS

As far as BMI-SDS is concerned, at diagnosis females showed a lower mean BMI-SDS than males (Table 2). During follow-up, in the whole population, BMI-SDS increased significantly both during treatment and after it until the achievement of FH. After the end of chemotherapy the variation in BMI SDS significantly differed between males and females (BMI SDS FH – BMI SDS EoT: -0.32 ± 1.23 vs. 0.24 ± 1.34 SDS, respectively, p 0 · 023): males decreased significantly their BMI-SDS (FH vs. EoT: p 0.047), whereas females demonstrated no improvement in BMI SDS (FH vs. EoT: p 0.210) (Figure 2). Therefore, at FH only females showed a significant increase of BMI-SDS than at sT (Table 2).

### Age of exposure to chemotherapy

According to age, 47 children were younger than four years at diagnosis of ALL (median age 3.06; range 1.20 – 3.89 years). Other 60 presented at sT a median age of 7.79 years (range 4.15 – 13.73 years). There was no significant difference between groups in sex distribution. During chemotherapy, a significant reduction of height-SDS was registered in both groups and it still persisted at FH (Table 3). BMI-SDS increased significantly from sT to EoT in both group, but it was still increased in comparison to the previous at sT only among children exposed to chemotherapy at a younger age (Table 3).

**Table 3:** **Variation of anthropometric data in patients according to age (younger or older than 4 years)**

	< 4 years	≥ 4 years
**Height SDS**		
**sT**	0.54 ± 1.06	0.67 ± 1.03
**EoT**	0.14 ± 1.13 (0.000)	0.34 ± 1.05 (0.003)
**FH**	0.08 ± 1.13 (0.001)	0.26 ± 1.14 (0.004)
**BMI SDS**		
**sT**	-0.03 ± 1.20	0.30 ± 1.40
**EoT**	0.48 ± 1.42 (0.005)	0.65 ± 1.29 (0.023)
**FH**	0.49 ± 0.97 (0.005)	0.53 ± 1.08

Only significant p-values versus sT are expressed into brackets.

### Multiple regression analysis

Multiple regression analysis identified TH and height SDS at sT as independent predictive factors for FH (coeff. 0.04, SE 0.02; coeff. 0.46, SE 0.12, respectively). Height SDS together with BMI SDS at sT was also identified as independent predictive factors for final BMI SDS (coeff. 0.30, SE 0.11; coeff. 0.41, SE 0.12, respectively).

## Discussion

According to our knowledge, this is the first study examining long-term growth outcomes in a wide cohort of un-irradiated Italian survivors from childhood ALL.

### Changes with time in height-SDS

Growth impairment has already been reported as a frequent complication of the treatment of ALL [17]. Patients treated when younger than four years and who received cranial irradiation seem to be the more affected [18, 19]. Nevertheless, a variety of other factors can contribute to growth impairment, including decreased nutritional intake, psychosocial dysfunction, chemotherapy and steroid therapy [20]. Data on long-term growth in children treated with chemotherapy alone are scarce and discordant [9, 12, 13, 18, 21–26].

Our data demonstrate that chemotherapy minimally affected final height, confirming some short-term previous published findings [18, 24, 25, 27]. In 2013, Vandecruys and colleagues described the longitudinal growth of 67 adult survivors of childhood ALL, treated from 1983 to 1989 according to European Organization for Research and Treatment of Cancer 58831/2 protocols with chemotherapy as the only treatment modality, and demonstrated a decrease in FH SDS from the time of diagnosis of -0.53 SDS in the standard-risk ALL group (45 patients) and of -0.73 SDS in the medium- or high-risk survivors (22 patients) [26]. In our study, survivors showed at FH a significant mean height loss of – 0.42 SDS (p 0 .000) compared to height SDS at diagnosis and, analyzing height-SDS adjusted according to TH, the loss in SDS from sT to FH still persisted (-0.30; p 0.000). Differences could be due to the inclusion in our study of a larger number of survivors treated on more recent chemotherapy protocols. The damage induced by chemotherapy could impede a complete catch-up growth, even if our survivors reached a FH within the normality. In fact, only TH and height-SDS at sT directly correlated with FH and they were both identified as independent predictive factors for FH in the multiple regression analysis, demonstrating than genetic potentiality together with growth pattern before ALL still remain the two main factors influencing FH.

In irradiated population, girls typically experienced greater growth impairment than boys [11, 28]. Also in our study, analyzing data adjusted according to TH, only females showed a significant persistent reduction of height-SDS data, while males seemed to partially catch up after the end of chemotherapy. Because of the retrospective design of our study and the lack of a description of pubertal data, we could only hypothesized that it is possibly due to the early occurrence of puberty in female survivors of childhood ALL but not in males, as already described in literature [29, 30].

How chemotherapy can affect growth is still unclear, even because hypothalamic-pituitary cells together with liver and chondrocyte do not have a high multiplicative capacity and, therefore, they have not usually been considered vulnerable to the action of chemotherapeutic agents. A reduction of growth hormone (GH) secretion induced by chemotherapy could be supposed. Rose and colleagues documented a hypothalamic-pituitary abnormality after chemotherapy in 83% of 18 hematological malignancy survivors (17 ALL) who were referred to their Clinic because of slow growth [31]. GH deficiency developed in 9 survivors. Even if this report did not represent a study of prevalence, authors hypothesized that chemotherapy alone may affect hypothalamic neurons through the loss of hypothalamic releasing or inhibitory hormone functions or through an altered processing of pituitary hormone. Host factors which might be associated with greater susceptibility to chemotherapy could be considered contributing factors [31]. On the contrary, Vandecruys and colleagues evaluated GH status in their population through glucagon stimulation test at the end of chemotherapy. In the 28% of cases, they demonstrated a decrease of GH secretion. This biochemical status did not correlate with the reduction in height SDS from diagnosis or end of treatment to FH [26]. Moreover, patients with an altered peak GH level at the end of chemotherapy were retested at the onset of puberty and demonstrated a normalized response to the GH stimulation test [26]. In older studies, a condition of GH-deficiency requiring GH replacement therapy has reported prevalence of 0.9 and 1.2% of childhood survivors of ALL treated with chemotherapy alone [32, 33]. In our study, data were collected retrospectively and we did not investigate GH secretion in our population. Nevertheless, the demonstration of limited growth during chemotherapy could support the hypothesis that GH secretion could be directly and acutely altered by chemotherapeutic agents and that, in acute phases, both ALL and its treatment can caused severe catabolic effects in children, leading to increasing protein breakdown and reducing protein synthesis. The growth-promoting and metabolic actions of GH are mediated by specific GH receptors. The absence of or reduction in functional proteins might result in a condition of partial, transient and reversible GH insensitivity [34]. All our patients were exposed to high dose, intravenous MTX associated to its intrathecal administrations and to glucocorticoids in pre-induction, induction and re-induction phases (Table 1). Mild late effects on growth could be due to a direct, structural negative effect of chemotherapy on epiphysis with partial recovery [35]. Up to now, we can demonstrate a significant and not obvious role of chemotherapy in long-term growth impairment, but we can only hypothesize a synergic effect of all chemotherapeutic compounds in altering the multitude and the functionality of cells in growth plate.

### Changes with time in BMI-SDS

Obesity is probably the most worrisome and common late effect in survivors of childhood ALL [36]. Razzouk in 2007 documented that 33.7% of 101 young adults treated previously with chemotherapy-alone for ALL were obese [37]. In addition, children treated for childhood cancer may be at unusually high risk from the consequences of obesity, particularly cardiovascular and metabolic comorbidities. Our data help in clarifying changes in BMI SDS over time, meanwhile and after chemotherapy for ALL. Excess weight gain usually starts soon after diagnosis, particularly during the first year of therapy, and it seems to be maintained [20, 38]. Nevertheless, up to now, long-term BMI data in an unirradiated population are not yet fully described and short-term data are extrapolated from small populations [39]. The rate of weight gain varies between studies [10, 37, 40–42]. In our study, BMI-SDS increased significantly from the start of treatment and the increase persisted until the achievement of FH. Increased energy intake and reduced habitual physical activity are commonly considered the main responsible factors of weight gain. During treatment, patients usually undergo changes in their lifestyle. The loss of physical activity may start during hospitalization or unwellness period of the patient, but it persists into adulthood due to a number of factors, including diminished exercise capacity, impaired motor function, diminished interest in recreational activity and over-protectiveness of the child’s primary caregivers [36]. In adulthood, almost 44% of ALL survivors are unlikely to meet the Centers for Disease Control and Prevention Recommendations for physical activity and over 74% are less likely to be physically active [43] and their dietary intake is not concordant with specific dietary recommendation [44]. In our study, BMI SDS at sT is identified as independent predictive factor for BMI-SDS at FH. This might indirectly document that, independently from the diagnosis of ALL and its treatment, environmental factors as a negative lifestyle acquired in the family setting during first years of life may intervene in the development of obesity during and after the treatment of ALL.

Exposure to chemotherapeutic medications seemed to differ between genders: females seemed more sensitive to effects of chemotherapy and they might be more at risk to present greater and more persistent metabolic alterations than males. Up to now, none of therapeutic choices and/or doses could explain this sexual dimorphism. Others factors should be considered. Female survivors are already known to be less active and to have a more unbalanced caloric intake than males [45]. Nevertheless, a definitive explanation of gender dymorphism could be identified only when other risk factors responsible for an indirect damage of the hypothalamus-pituitary region or of the metabolic pathways will be studied [36]. Our findings confirm and reinforce recent Harper’s data [27, 46]. The group from Cambridge presented data from only 27 ALL survivors (17 females) who reached FH. Only in females, persisting increases in weight-SDS and BMI-SDS (+1.45 vs. +0.46, p < 0.0001) were evident at FH [27].

In our cohort, age at diagnosis did not correlate with final BMI SDS and it was not identified as its independent predictive factors. Nevertheless, examining population according to age-subgroups (younger and older than 4 years at sT), only younger children presented a persistent increase of BMI SDS after the end of chemotherapy. This might support that chemotherapy acts similarly to radiotherapy in altering body composition later-in-life via blunting hypothalamic leptin sensitivity and via modification of metabolism.

## Conclusions

In conclusion, our study demonstrates that chemotherapy alone, as in AIEOP protocols, minimizes the loss in FH SDS together with the increase in BMI SDS in adult survivors of childhood. These results could be positively influenced by the fact that, in all the included Italian centers, the management of oncologic patients involves pediatric endocrinologists from the early stages of treatment, allowing early evaluation of pathological conditions and, consequently, early interventions. Nevertheless, both males and females experienced a loss in height-SDS during chemotherapy that was not followed by an appropriate catch-up growth, and an increase of BMI-SDS. Physicians have to keep in mind that cancer survivors, because of treatment (potential cardiotoxic chemotherapy), are prone to develop cardiovascular disease. Obesity can increase the risk for morbidity and mortality due to diabetes, coronary artery disease, atherosclerosis and other diseases related to overweight in adulthood [38], especially in females [47]. Our findings confirmed females as more at risk of either becoming or persisting obese long after chemotherapy. This enables us to focus our efforts in preventing their weight gain and long-term complications of obesity. Nevertheless, the majority of adult survivors are followed by primary health care physicians, rather than by an oncologic center. This leads to an imperative need: educate all health workers on preventing these risks and on developing specific intervention strategies, including correct dietary and lifestyle choices [48] in ALL survivors, even if treated with chemotherapy only.

## Abbreviations

ADM: Adriamycin
AIEOP: Italian Association of Pediatric Hematology and Oncology
ALL: Acute Lymphoblastic Leukemia
ARA-C: Cytosine arabinoside
BMI: Body Mass Index
CPM: Cyclophosphamide
DESA: Dexamethasone
DNM: Daunorubicin
EoT: End of Treatment
FH: Final Height
GH: Growth Hormone
IFO: Ifosfamide
IT: Intrathecal
IV: Intravenously
L-ASP: L-asparaginase
MTX: Methotrexate
PDN: prednisolone
SD: Deviation Score
SDS: Standard Deviation Score
sT: diagnosis of ALL
TH: Target Height
VCR: Vincristine
VM-26: teniposide
WBC: White Blood Cells
6-MP: 6-mercatopurine
6-TG: 6-thioguanine.
